# Cardiac Complications Associated With COVID-19 Vaccination: A Systematic Review of Cohort Studies

**DOI:** 10.7759/cureus.78535

**Published:** 2025-02-05

**Authors:** Indrajit Banerjee, Jared Robinson, Indraneel Banerjee

**Affiliations:** 1 Pharmacology, Sir Seewoosagur Ramgoolam Medical College, Belle Rive, MUS; 2 Surgery, Sir Seewoosagur Ramgoolam Medical College, Belle Rive, MUS; 3 Urology, Penn Highlands Healthcare, Pennsylvania, USA

**Keywords:** 2019-ncov vaccine, ad26covs, bnt162 vaccine, covid-19 vaccines, mrna-1273, myocarditis

## Abstract

The COVID-19 global pandemic affected every human on earth, and we are still currently feeling the repercussions. The unprecedented transmission of the virus and the response as well as the mobilization of the major health authorities internationally resulted in one of the largest-scale immunization drives in modern history. As of January 16, 2025, 13.64 billion COVID-19 vaccines have been administered globally. Cardiac adverse effects, such as the development of pericarditis and or myocarditis after receiving the COVID-19 vaccine, have been a major focus of study. In most systematic reviews reported globally, evidence was synthesized from case reports and case series. This systematic review aims to amalgamate the data from various cohort studies to identify the risk of the development of adverse effects after the COVID-19 vaccine. An extensive review of the literature was done on the following databases: PubMed, Cochrane Central Register of Controlled Trials (CENTRAL), Trip database, and Google Scholar. All cohort studies included were completed and available between December 1, 2020 and December 31, 2024 and were based on the cardiac adverse effects from the COVID-19 vaccinations. A total of 18,272 articles were screened initially. Four studies were finally assessed regarding the cardiac side effects of the COVID-19 vaccinations and were ultimately included in the systematic review based on inclusion and exclusion criteria.

Immunization with an mRNA-based COVID-19 vaccine may directly cause cardiovascular adverse events such as the development of myocarditis or pericarditis. The likelihood of such an event occurring is minimal but is most certainly a possibility, the risks of such adverse effects are notably raised in younger males between the ages of 16 and 39 years in age receiving their second dose of an mRNA-based vaccine. It is thus advised that those individuals who fall into the above category be labeled as “higher risk” and should have increased post-vaccination surveillance and follow-up to earlier diagnose the development thereof. The benefits of the vaccine still do, however, by far outweigh the minimal risks involved and it is thus advised that immunization effort continues in earnest.

## Introduction and background

The COVID-19 global pandemic affected every human on earth, and we are still currently feeling the repercussions of [1]. The unprecedented transmission of the virus and the response as well as the mobilization of the major health authorities internationally resulted in one of the largest-scale immunization drives in modern history [2]. It is currently difficult to conceptualize that no specific vaccine against the SARS-CoV-2 virus existed during the initial outbreak of the pandemic and it took only 11 short months for the first vaccine against the COVID-19 virus to be developed, tested, and receive emergency use authorization [3]. The pooling of global resources, e.g. financial, technological, and scientific collaboration, allowed for more than one type of vaccine to be developed within a short time frame. The majority of the vaccines were developed with the use of mRNA technology, e.g., Pfizer-BioNTech (New York, NY) and Moderna (Cambridge, MA) vaccines, while a few used killed components for their synthesis, mRNA technology allows for a more targeted approach as well as a more rapid scale-up of vaccine production [4].

The global immunization campaign undoubtedly saved an innumerable number of lives. It did, however, receive an immense backlash and criticism from various groups who completely denied and vehemently opposed the use of the vaccine, a study published in the Journal of Paediatric Child Health claims that the vaccines prevented 14.4 million deaths [5]. The fear of the speed at which these novel vaccines had been developed and the potential for unknown “detrimental side effects” such as lowered immunity, sterility, and/or cancer to develop further fueled the skepticism and only entrenched those on their “no jab” position [6].

As with any vaccination against infectious diseases, adverse effects are possible. Cardiac adverse effects, such as the development of pericarditis and or myocarditis after receiving the COVID-19 vaccine, have been a major focus of study. In most systematic reviews reported globally, evidence was synthesized from case reports and case series [7]. This systematic review aims to amalgamate the data from various global cohort studies of large sects of the population, for the risk of the development of such adverse effects after the COVID-19 vaccine to be quantified. This study is vital as it does not rely on mere anecdotal evidence, but combines multiple high-level controlled studies, allowing its findings to be accurate.

This paper with preliminary findings was presented at the Eighth International Symposium of New Frontiers in Cardiovascular Research, Koç University Hospital, Istanbul, Turkey on September 26-27, 2024 by Indrajit Banerjee.

## Review

Methodology

The Preferred Reporting Items for Systematic Reviews and Meta-Analyses (PRISMA) 2020 guidelines were employed throughout the conduction of this systematic review.

Literature Searches

An extensive review of the literature was done on the following databases: PubMed, Cochrane Central Register of Controlled Trials (CENTRAL), Trip database, and Google Scholar. Medical subject headings (MeSH) term combinations were used for data synthesis ((((((COVID-19 Vaccines) OR (BNT162 Vaccine)) OR (2019-nCoV Vaccine mRNA-1273))) OR (Ad26COVS1)))) AND (Complications))))).

Inclusion Criteria

All cohort studies included were completed and available in the following databases, viz. PubMed, Cochrane Central Register of Controlled Trials (CENTRAL), Trip database, and Google Scholar, between December 1, 2020 and December 31, 2024 were based on the cardiac adverse effects from the COVID-19 vaccinations. These studies were thoroughly screened and were subsequently included in this systematic review. Full-text cohort studies published in English were both identified and incorporated into this systematic review.

Exclusion Criteria

The exclusion of any of the cohort studies identified was subject to the availability of the data concerning the side effects noted after the administration of the COVID-19 vaccines. Randomized clinical trials (RCTs), non-randomized clinical trials (NRCTs), case-control studies, cross-sectional studies, abstracts, case studies, reports, editorials, viewpoints, case series as well as letters to the editor/correspondence manuscripts were rejected from this systematic review.

Data Synthesis

Data synthesis was intimated through a primary screening of the relative titles and their abstracts. After which full texts of the examined titles of the cohort studies that met the eligibility requirements were considered for the final selection. The literature evaluation was performed independently by Jared Robinson and Indraneel Banerjee to ensure objectivity. The extracted data was validated by the third senior researcher Indrajit Banerjee. The extracted data included the study authors, year, country, design, age group, inclusion criteria, study population, vaccine type, total cases of myocarditis/pericarditis, the total number vaccinated, the study's main findings, the vaccines implicated, and the gender most implicated (Table 1).

**Table 1:** Various databases used

Databases searched	Boolean Operators and Keywords	Total number of articles
PubMed	(((((COVID-19 Vaccines) OR (BNT162 Vaccine)) OR (2019-nCoV Vaccine mRNA-1273)) OR (Ad26COVS1)) AND (Complications)) OR (Pericarditis) Filters: from 2020 - 2024	6,802
Cochrane Central Register of Controlled Trials (CENTRAL)	(((((COVID-19 Vaccines) OR (BNT162 Vaccine)) OR (2019-nCoV Vaccine mRNA-1273)) OR (Ad26COVS1)) AND (Complications)) OR (Pericarditis) in Title Abstract Keyword	614
Google Scholar	COVID-19 Vaccines OR BNT162 Vaccine OR 2019-nCoV Vaccine mRNA-1273 OR Ad26COVS1 AND Complications OR Pericarditis Custom range: 2005-2024	9,900
Trip	COVID-19 Vaccines OR BNT162 Vaccine OR 2019-nCoV Vaccine mRNA-1273 OR Ad26COVS1 AND Complications OR Pericarditis from_date:2005 to_date:2024	956
Total		18,272

Results

**Figure 1:**
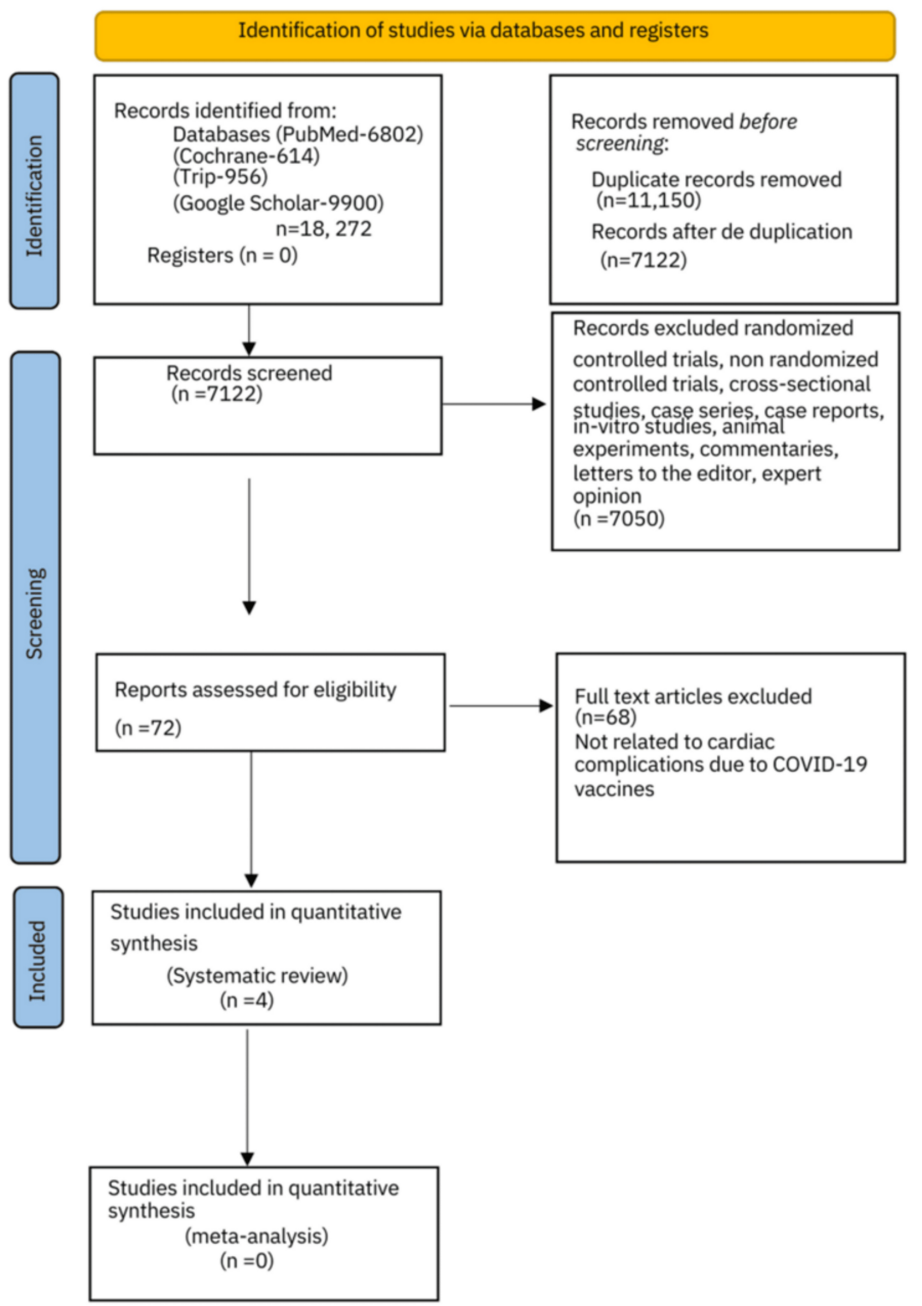
PRISMA 2020 flow chart

The literature search conducted produced 18,272 articles. Among these, 11,150 were noted as duplicates and excluded from the initial analysis. Thus, 7,122 manuscripts were screened after deduplication. Abstracts, case studies, reports, editorials, viewpoints, cross-sectional studies, randomized control trials, non-randomized controlled trials, case-control studies, case series, and letters to the editor/correspondence manuscripts (n=7,050) were additionally excluded. A total of 72 full-text articles were assessed for eligibility. An in-depth evaluation and analysis further excluded 68 articles from the analysis quality assessment based on inclusion and exclusion criteria. Four cohort studies were finally assessed regarding the cardiovascular side effects of the COVID-19 vaccinations and were ultimately included in the systematic review (Figure 1).

Quality Assessment and Critical Appraisal

Newcastle Ottawa Scale was used for the quality assessment of the cohort studies. Quality assessment was done based on three domains namely the selection (maximum four stars), comparability (maximum two stars), and outcome (maximum three stars). A total score of 9 was allotted for each study. A score of minimum 7/9, i.e., >70% and above was included in this study for analysis (Table 2).

**Table 2:** Newcastle Ottawa Scale for quality assessment of the selected cohort studies

Author	Selection (maximum 4 stars)				Comparability (maximum 2 stars)	Outcome (maximum 3 stars)			Overall score
	Representativeness of the exposed cohort	Selection of the non-exposed cohort	Ascertainment of exposure	Demonstration that outcome of interest was not present at start of study	Comparability of cohorts on the basis of the design or analysis	Assessment of outcome	Was followed up long enough for outcomes to occur	Adequacy of follow up of cohorts	
Buchan et al. [8]	*	*	*	*	*	*	*	*	8
Simone et al. [9]	*	*	*	*	*	*	*	*	8
Mevorach et al. [10]	*	*	*	*	*	*	-	*	7
Karlstad et al. [11]	*	*	*	*	**	*	*	*	9

Tables 3, 4 depict the study authors, year, country, design, age group, inclusion criteria, study population, vaccine type, total cases of myocarditis or pericarditis, the total number vaccinated, the study's main findings, the vaccines implicated, and the gender most implicated.

**Table 3:** Summary of the studies, duration, age group, and inclusion criteria

Author, year	Country	Duration	Design	Age group	Inclusion criteria
Buchan et al., 2022 [8]	Canada	10 months	Population based-cohort study	12-81 years	Every individual in the Ontario region who received a minimum of 1 dose of the mRNA COVID-19 vaccine between the 14th of December 2020 to the 4th of September 2021.
Simone et al., 2021 [9]	United States of America	8 months	Population based-cohort study	18 years and older	Cases were identified via the (KPSC) Regional Immunization Practice Committee. Those who were hospitalized within 10 days of the administration of the vaccine and whose discharged diagnosis was found to be myocarditis.
Mevorach et al., 2021 [10]	Israel	6 months	Retrospective cohort study	16 years and older	The diagnostic criteria for myocarditis were adapted from the case definition and classification of the Brighton Collaboration.
Karlstad et al., 2022 [11]	Sweden, Finland, Norway, and Denmark	11 months	Cohort study	12 years or older	Nordic residents 12 years and older who were vaccinated and followed up from December 27, 2020 up until the development of myocarditis or pericarditis or October 5, 2021.

**Table 4:** Vaccine type, total cases of myocarditis, total number vaccinated, findings, vaccines implicated, and gender IRRs: incidence rate ratios

Author, year	Vaccine type	Total cases of myocarditis/pericarditis	Total number vaccinated	Main findings	Vaccines implicated	Gender implicated M/F
Buchan et al., 2022 [8]	mRNA	297	19,740,741	69.7% of the cases of pericarditis/ myocarditis occurred on the second dosage. The highest rate of myocarditis/pericarditis was noted in Males aged 18 to 24 years.	mRNA-1273 as the second dose 299.5 cases per 1,000,000 doses; 95% CI (171.2-486.4 cases per 1,000,000 doses). BNT162b2 as the second dose was 59.2 cases per 1,000,000 doses (95% CI, 19.2-138.1 cases per 1,000,000 doses)	M
Simone et al., 2021 [9]	mRNA	15 (2 after the first dose and 13 after the second)	2,392,924	An incidence of 0.8 cases per 1 million (on the first dose) and a greater 5.8 cases per 1 million occurred with the second dose.	mRNA-1273 = 50.2% of the doses; BNT162b2 = 50% of the doses	M
Mevorach et al., 2021 [10]	mRNA	304	9,289,765	Overall the risk difference between the 1st and 2nd doses of the vaccine was 1.76 per 100,000 persons, 95% CI, 1.33 to 2.19. For males the overall risk difference was 3.19 (95% CI, 2.37 to 4.02). As compared to the 0.39 (95% CI, 0.10 to 0.68) among the females.	BNT162b2 mRNA vaccine	M
Karlstad et al., 2022 [11]	mRNA (adenoviral DNA vaccine)	1,149	23,122,522	The second dose was associated with higher risk of myocarditis, with adjusted IRRs of 1.75 (95% CI, 1.43-2.14) for BNT162b2 and 6.57 (95% CI, 4.64-9.28) for mRNA-1273.	BNT162b2, mRNA-1273 and AZD1222	M

Discussion

As of January 16, 2025, 13.64 billion COVID-19 vaccines have been administered globally. The first COVID-19 vaccine to be administered outside of a clinical trial setting was developed by Pfizer/BioNTech and was done so on December 8, 2020. The unprecedented scale of the vaccine drive cemented the immunization campaign against the COVID-19 pandemic as one of the largest in the history of mankind [12,13].

The development of the COVID-19 vaccine occurred over a remarkably short time frame of 11 months, after which it was given emergency use status and intensive post-marketing surveillance was initiated. This short synthesis period is largely owed to the unification of the global health effort with the pooling of international data and knowledge as multiple health agencies and governments banded together in order to share information and data to synthesize the vaccine in such a short period [2]. As with any medical intervention, the possibility of unforeseen adverse effects is a reality. The staggering figure of 13.64 billion doses being administered to date with the super-added enhanced pharmacovigilance due to the novelty of the vaccines, naturally ensured and only increased the likelihood of any potential adverse reactions or side effects being reported and identified [13].

The intense and hypervigilant media and reporting surrounding any of the post-immunization side effects were double-edged. It provided unprecedented feedback to the WHO as well as other health regulatory authorities which still deemed the vaccine as “safe” [14]. However, the downside to the massive media coverage thereof, was the fact that immunization side effects and post-vaccine reactions were largely reported and shared on forums that had either no or little scientific basis or proof, thus driving and further strengthening the plight of those either in fear or anti-the-vaccine [15].

This systematic review has incorporated and reviewed both the data and findings of five population cohort studies, which have primarily focused on the cardiovascular complications most specifically: the development of myocarditis and pericarditis after receiving the COVID-19 vaccine. This study was performed using proven scientific processes, i.e., the systematic review methodology and the true likelihood of such side effects and the population in which most plagues have been determined [8-12].

In totality, the four cohort studies in our systematic review. In general, our study proved that the majority of cases of myocarditis/pericarditis occurred in the male gender. According to the study by Mevorach et al. included in our systematic review, the overall risk difference for males was 3.19 (95% CI, 2.37 to 4.02). As compared to the 0.39 (95% CI, 0.10 to 0.68) among the females [10]. This is in alignment with a fellow systematic review performed by Knudsen et al., who concluded that males were the gender more predisposed to developing myocarditis/pericarditis. Furthermore, the findings between the two studies were similar as the age group most affected were those under 40 years of age and were immunized with an mRNA vaccine [16].

The second dose of the vaccine was proven to attenuate myocarditis/pericarditis more so than the first. According to the Nordic cohort study including Sweden, Norway, Finland, and Denmark performed by Karlstad et al., which was included in our systematic review, the second dose was associated with a higher risk of myocarditis, with adjusted incidence rate ratio (IRR) of 1.75 (95% CI, 1.43-2.14) for BNT162b2 and 6.57 (95% CI, 4.64-9.28) for mRNA-1273 [11]. A controlled case series study, performed by Stowe et al. showed that the highest relative incidence was noted after the second dose at 5.34 (95% confidence interval (CI) (3.81, 7.48); p < 0.001) for BNT162b2 and 56.48 (95% CI (33.95, 93.97); p < 0.001) for mRNA-1273 [17].

The cardiovascular side effects noted in this study were predominantly a cause of the mRNA-based vaccine and most commonly occurred after the administration of the second dose in all ages, the cohort study included in this systematic review conducted by Patone et al. concluded that no correlation was noted with the ChAdOx1 vaccination and the development of myocarditis/pericarditis. Furthermore, this study concurred with the above notion that there was an increased risk of the development of such cardiovascular events after the second immunization event as opposed to the first [12].

The cardiovascular adverse effects of myocarditis and pericarditis may be associated with the COVID-19 vaccine. The likelihood of such an event occurring is minimal but is most certainly a possibility, the risks of such adverse effects are notably raised in younger males between the ages of 16 and 39 years receiving their second dose of an mRNA-based vaccine.

This study could be further improved by, taking into consideration the global infection rate of the study populous in order to try and delineate whether the myocarditis/pericarditis was a direct result of the infection with SARS-CoV-2 virus or of the vaccine to establish a more accurate risk profile. Furthermore, the results could be improved via the stratification of each case of myocarditis or pericarditis into a degree of its severity with the timeline in which it occurred post-vaccination.

Limitation

Full-text cohort studies published in English were both identified and incorporated into this systematic review. Subsequently, manuscripts published in other languages were excluded was the limitation of this study.

## Conclusions

Immunization with an mRNA-based COVID-19 vaccine may potentially have an association with cardiovascular adverse events such as the development of myocarditis or pericarditis. The likelihood of such an event occurring is minimal, the risks of such adverse effects are notably raised in younger males between the ages of 16 and 39 years in age receiving their second dose of an mRNA-based vaccine, but the risks do not outweigh the benefit of receiving the vaccination. It is thus advised that those individuals who fall into the above category be labeled as “higher risk” and should have increased post-vaccination surveillance such as symptomatic monitoring, routine ECGs, and regular follow-ups so as to earlier diagnose the development thereof. The benefits of the vaccine still do however, by far outweigh the minimal risks involved and it is thus advised that immunization effort continues in earnest on a global scale whilst particularly targeting the elderly and immunocompromised. Government agencies must be open and honest with the populous to prevent misinformation, as ultimately the vaccination effort has saved over 14 million lives.
